# Factors Influencing Nurses' Work Excitement and Frustration During a Pandemic (COVID‐19): A Cross‐Sectional Study

**DOI:** 10.1002/nop2.70397

**Published:** 2025-12-15

**Authors:** Alireza Houshangi, Ali Mohammadabadi, Fateme Noghani, Shima Haghani

**Affiliations:** ^1^ Department of Nursing, School of Nursing and Midwifery Tehran University of Medical Sciences Tehran Iran; ^2^ Department of Nursing, School of Nursing and Midwifery Sabzevar University of Medical Sciences Sabzevar Iran

**Keywords:** COVID‐19, excitement, frustration, nurses

## Abstract

**Aim:**

To investigate the relationship between work excitement and work frustration among nurses during the COVID‐19 pandemic and to identify individual and occupational factors associated with these emotional states.

**Design:**

Cross‐sectional study.

**Methods:**

A cross‐sectional study was conducted among nurses working in COVID‐19 care centers in Iran. A total of 265 nurses were included in the study, which exceeded the minimum required sample size (*n* = 163) calculated based on power analysis. Data were collected using self‐report questionnaires assessing individual and occupational characteristics, work excitement, and work frustration.

**Results:**

A significant negative correlation was found between work excitement and work frustration. Occupational factors such as fixed shifts, incentive bonuses, and working in intensive care units were associated with increased work excitement and decreased work frustration. Conversely, excessive workload, irregular shifts, and inadequate staffing levels were linked to higher levels of work frustration. Individual factors, including marital status, parental status, and housing status, also influenced work‐related emotions. Married nurses with children and homeowners reported higher levels of work excitement and lower levels of work frustration. Male nurses and those with higher incomes also experienced increased work excitement and decreased work frustration.

**Patient or Public Contribution:**

No patient or public contribution.

## 1 Introduction

The COVID‐19 pandemic has underscored the pivotal role of nurses as frontline healthcare workers (Kumar 2022). As they grappled with unprecedented challenges, including overwhelming patient loads (Zamanzadeh et al. 2021), resource scarcity (Zamanzadeh et al. 2021), and the constant threat of infection, nurses exhibited remarkable resilience and dedication (Batassini and Beghetto 2024; Muhammad et al. 2023). However, the emotional toll of this crisis has been profound, with many nurses experiencing heightened levels of stress, anxiety, and burnout (Mao et al. 2024).

While the negative consequences of the pandemic on nurses' well‐being have been explored in different studies (Zamanzadeh et al. 2021; Muhammad et al. 2023; Mao et al. 2024), a more nuanced understanding of the factors affecting work‐related emotional experiences of nurses during this time is needed. This study seeks to delve into the interplay between work excitement and work frustration, two key work‐related emotional states, and the factors that influence them.

Work excitement is a positive emotional state characterised by enthusiasm, eagerness, and a deep commitment to one's job (Chang et al. 2019). It's more than just liking your work; it's a passionate engagement that fuels motivation, creativity, and a proactive approach to challenges (Chang et al. 2019, 2014).

Work excitement is often sparked by engaging in challenging and diverse tasks, experiencing professional growth, and feeling valued and supported in the workplace (Chang et al. 2019, 2014). It's a positive force that can enhance job performance, increase job satisfaction, and reduce burnout (Santos et al. 2021; Moulton et al. 2022; Sadovich 2002). Critical conditions such as the COVID‐19 crisis can lead to increased occupational stress and work pressure, potentially fostering feelings of frustration among nurses (Smart et al. 2024; Iddrisu et al. 2023). Work frustration is a negative emotional state that arises when an individual encounters obstacles that hinder them from achieving their professional goals or fulfilling their work‐related needs (Busque‐Carrier et al. 2022). These obstacles can be both external, such as challenging work conditions or limited resources, and internal, such as a mismatch between personal values and job requirements (Busque‐Carrier et al. 2022).

Previous studies have examined some factors related to work excitement and frustration in various professions, but there is a dearth of research specifically focused on nurses, particularly during a pandemic. Chang et al. (2019) found that work excitement was positively influenced by organisational commitment, job challenge, and a positive violence prevention climate for nurses. Similarly, Adinata (2023) demonstrated a correlation between individual characteristics, such as values and work preferences, and work excitement. On the other hand, research has consistently shown that work frustration is associated with increased job demands, decreased job control, and a lack of organisational support. For example, Garces‐Cabanas and Dano (2022) identified challenging experiences, lack of control, and feelings of undervaluation as key factors contributing to work frustration. While these studies provide valuable insights, they do not fully address the unique context of the COVID‐19 pandemic.

This study aims to address this gap in the literature by investigating the factors associated with work excitement and frustration among nurses during the COVID‐19 pandemic. Specifically, we will explore how individual factors (e.g., age, gender, education) and occupational factors (e.g., workload, income and Patient‐to‐Nurse Ratio) influence these emotional states. Additionally, the study will examine the relationship between these two emotional states. By examining these factors, we seek to gain a deeper understanding of the complex interplay between individual characteristics and organisational contexts that shape nurses' emotional experiences.

By identifying factors related to work excitement and work frustration, healthcare organisations can implement strategies to foster a more positive and supportive work environment for nurses.

Based on the identified gap in the literature, this study will address the following research questions:

1. What are the individual and occupational factors associated with work excitement among nurses during the COVID‐19 pandemic?
2. What are the individual and occupational factors associated with work frustration among nurses during the COVID‐19 pandemic?
3. Is there a relationship between work excitement and work frustration among nurses during the COVID‐19 pandemic?

## 2 Methods and Materials

### 2.1 Study Design

A cross‐sectional study design was employed to examine work excitement and work frustration among nurses working in five hospitals selected through cluster sampling from COVID‐19 care centres affiliated with Semnan University of Medical Sciences. Data were collected in autumn 2021.

### 2.2 Sample Size Calculation

A power analysis was conducted to determine the minimum sample size required to estimate ‘work excitement’ with a 95% confidence level and a margin of error of 1.7 (equivalent to 10% of the minimum scale score). Based on Schmidt et al.'s findings, an estimated standard deviation of 11.06 was used. Using the following formula, the calculated minimum sample size was 163 participants.

$$\begin{array}{c} n=\frac{z_{1-\frac{\alpha }{2}*}^{2}{\mathrm{SD}}^{2}}{d^{2}}=\frac{1.96^{2}\times 11.06^{2}}{{\left(1.7\right)}^{2}}=162.53\approx 163 \end{array}$$

A similar calculation was performed for ‘work frustration,’ resulting in a smaller required sample size. However, to ensure adequate statistical power for both constructs, a sample size of 163 participants was adopted for both variables.

### 2.3 Participants and Sampling

A two‐stage sampling procedure was used to recruit participants from Semnan Province. In the first stage, four cities were selected from the list of all cities in the province through simple random sampling using a random number table. In the second stage, to account for heterogeneity in population size and healthcare infrastructure, the four selected cities were considered as five clusters (one city contributing two clusters). One hospital was then selected from each cluster by simple random sampling using a random number table, yielding five hospitals in total. From each selected hospital, 100 nurses were invited to participate using convenience‐based simple random sampling from the available nursing staff lists. However, 34% of the invited nurses (170 individuals) did not complete the questionnaires, and an additional 11.8% (59 individuals) had incomplete responses. After excluding these incomplete questionnaires, 271 complete responses remained.

To ensure equal representation across hospitals for cluster‐comparative analyses, the analytical sample was standardised to 53 participants per hospital. When a hospital had more than 53 complete responses, 53 questionnaires were selected through simple random sampling using a random number table; when a hospital had exactly 53 complete responses, all were included. This procedure resulted in a final analytical sample of 265 nurses. This adjustment did not affect the statistical power of the study, as the final sample size remained substantially above the minimum requirement of 163 participants determined by power analysis.

The non‐response rate (34%) and incomplete response rate (11.8%) may introduce a risk of non‐response bias. To minimise this risk, data collection was conducted across multiple hospitals and shifts to maximise diversity in participant availability. Follow‐up reminders were also provided to nurses who initially did not complete the questionnaire; however, the reported non‐response and incomplete response rates reflect the final numbers after these follow‐up efforts. Additionally, demographic and occupational characteristics of respondents were compared across the five hospitals, and no substantial differences were identified, reducing concerns about systematic non‐response bias.

To be eligible for inclusion in this study, participants were required to be registered nurses possessing a minimum of a bachelor's degree, with at least 6 months of clinical experience, including a minimum of 1 month of direct care for COVID‐19 patients (regardless of their specific job title). This criterion was implemented to ensure sufficient exposure to the unique stressors associated with caring for COVID‐19 patients. Participants were excluded from the study if they declined participation or provided incomplete questionnaire data.

### 2.4 Data Collection

Data for this study was gathered using four questionnaires: a demographic questionnaire, an occupational information questionnaire, the Work Excitement Scale, and the Work Frustration Scale. Prior to data collection, the face and content validity of the questionnaires were established through expert panel review by 10 faculty members in the field of nursing. Each item was evaluated for relevance, clarity, and simplicity. Based on their feedback, items were revised, removed, or reworded as necessary. Additionally, the Content Validity Ratio (CVR) and Content Validity Index (CVI) were calculated for each item to quantitatively assess content validity, with all items meeting the accepted thresholds (CVR ≥ 0.62, CVI ≥ 0.78).

#### 2.4.1 Demographic and Occupational Information Data Questionnaire

The researcher‐developed demographic and occupational information questionnaire assessed individual characteristics and key job‐related factors potentially influencing nurses' work experiences. Demographic items included age, gender, marital status, parenthood, education level, and housing status. Occupational variables covered employment type, work experience, shift work patterns, job title, unit, nurse‐to‐patient ratio, monthly working hours, income, and incentive bonus.

#### 2.4.2 Work Excitement and Work Frustration Scale

The Work Excitement (WEXCIT) instrument, developed by Simms et al. (1990), includes four subscales that assess different aspects of nurses' emotional experiences in the workplace. In the present study, we used two of these subscales: Work Excitement and Work Frustration. Both have demonstrated strong psychometric properties in previous research, with reported Cronbach's alpha values of 0.92 and 0.93, respectively (Erbin‐Roesemann 1995). The psychometric properties of this instrument have been consistently affirmed in subsequent research (Chang et al. 2019).

The Work Excitement subscale evaluates nurses' levels of enthusiasm and positive engagement with their work.

It comprises three components—General Enthusiasm and Excitement with Work, Excitement with Life, and Learning—and uses a 5‐point Likert response format ranging from strongly agree to strongly disagree (17 item, total score range: 17–85). Higher scores indicate greater work excitement.

The Work Frustration subscale measures the extent of frustration experienced by nurses in their work environment.

This subscale also includes three components—Inappropriate Utilisation of Resources, Work Arrangements, and Morale—and uses a 4‐point Likert scale ranging from “not at all” to “a lot” (24 items, total score range: 24–96). Higher scores reflect greater work frustration.

### 2.5 Ethical Considerations

Ethical approval for this study was obtained from the Tehran University of Medical Sciences under the code IR.TUMS.FNM.REC.1400.048.

All necessary permits were obtained before commencing research activities. Prior to initiating research, all necessary permissions were acquired. To uphold ethical principles and safeguard participant autonomy, potential participants received comprehensive information regarding the study's objectives, methodology, and data confidentiality measures. Participation was voluntary, and only those who provided informed consent were included in the study.

In accordance with copyright regulations, formal authorization to utilise and translate the Work Excitement (WEXCIT) for the purposes of this study was secured from the primary author, Ms. Marla Erbin‐Roesemann, on December 6, 2020, via electronic correspondence.

We followed the STROBE reporting guideline to ensure transparent and standardised reporting of this cross‐sectional study.

### 2.6 Data Analysis

Data analysis was performed using SPSS software, version 27. Qualitative data were summarised using frequency and percentage, while quantitative data were presented as mean and standard deviation. To investigate the correlation between individual and occupational factors and levels of work‐related excitement and frustration, we conducted independent samples t‐tests, ANOVA, and Pearson correlation analyses to compare group means and assess linear relationships. The Kolmogorov–Smirnov test was used to assess normality of continuous data. A significance level of 0.05 was set for all statistical tests.

## 3 Results

From the 500 nurses invited to participate, 271 accurately completed the questionnaires. To ensure equal representation across hospitals, data from 265 participants (53 from each hospital) were analysed. The missing data primarily involved participants who did not return the questionnaire (34%) or skipped some items (11.8%). No systematic differences were observed in demographic or occupational characteristics between respondents with complete and incomplete data, suggesting that the data were likely missing at random (MAR).

### 3.1 Demographic and Occupational Information of Participants

The sample comprised 56.6% females (*n* = 150) and 43.4% males (*n* = 115), with a mean age of 33.17 years. The majority of participants held a Bachelor of Science in Nursing (BSN) degree (86.4%). Most were married and had children. The average monthly workload was 207.5 h, with the majority working rotating shifts. Only 21.8% held supervisory positions (shift supervisors or head nurses). Most reported receiving an average salary (74%), and only 60% received performance‐based incentives. Nurses were primarily responsible for more than four patients per shift (73.6%). The majority possessed over 5 years of experience (61.1%) and held permanent positions (58.9%). Additionally, most owned their homes (52.8%), had health insurance (96.6%), and were employed in critical care units (CCU, ICU, Dialysis) or emergency units (52.4%). A detailed overview of this information is presented in Table 1.

**TABLE 1:** Demographic characteristics and occupational information of participants.

Demographic characteristics of participants		Occupational information of participants	
Variables	*n* (%)	Variables	*n* (%)
Gender		Employment status	
Male	115 (43.4)	Permanent	156 (58.9)
Female	150 (56.6)	Temporary	109 (41.1)
Marital status		Work experience	
Single	100 (37.7)	< 1	15 (5.7)
Married	145 (54.7)	1 to 5	88 (33.2)
Divorced	18 (6.8)	6 to 10	97 (36.6)
Widowed	2 (0.8)	> 10	65 (24.5)
Education level		Position	
Bachelor's degree	229 (86.4)	Clinical nurse	207 (78.1)
Master's degree	26 (9.8)	Shift supervisor	38 (14.3)
Doctorate degree	10 (3.8)	Head nurse	20 (7.5)
Parental status		Unit	
Not applicable (single)	100 (37.7)	Medical surgical unit	73 (27.5)
With children	122 (46.0)	Intensive care units	70 (26.4)
Without children	43 (16.2)	Emergency unit	122 (46.0)
Housing status		Average patient‐to‐nurse ratio in your unit	
Owned	140 (52.8)	< 3	24 (9.1)
Rented	60 (22.6)	3 to 4	46 (17.4)
Living with parents	65 (24.5)	5 to 8	84 (31.7)
		> 8	111 (41.9)
Insurance status		Incentive bonus	
Insured	256 (96.6)	Yes	159 (60.0)
Uninsured	9 (3.4)	No	106 (40.0)
Age		Income	
Mean (SD)	33.17 (8.36)	Less than average	48 (18.1)
		Average	196 (74.0)
		Higher than average	21 (7.9)
		Shift pattern	
		Fixed	33 (12.5)
		Rotating	232 (87.5)
		Monthly working hours	207.56 (45.30)
		Mean (SD)	

### 3.2 Hypothesis 1

There is a relationship between individual and occupational factors of nurses and their level of work excitement.

The mean work excitement score for all participants was 53.81 (SD = 12.97). To examine the relationship between dichotomous categorical variables and work excitement, independent samples t‐tests were conducted. As shown in Table 2, results indicated significant associations between shift pattern and receipt of incentive bonuses and work excitement (*p* < 0.05). Nurses working fixed shifts and those receiving incentive bonuses reported higher levels of work excitement.

**TABLE 2:** Work excitement scores and their relationship to demographic and occupational factors.

Variables	Mean (SD)	Variables	Mean (SD)
Gender		Employment status	
Male	55.46 (12.84)	Permanent	53.38 (14.14)
Female	52.54 (12.97)	Temporary	54.42 (11.12)
	*p* = 0.07*		*p* = 0.52*
Marital status		Work experience	
Single	56.60 (12.26)	< 1	49.26 (12.93)
Married	55.72 (13.15)	1 to 5	55.31 (11.07)
Divorced	56.55 (13.04)	6 to 10	53.55 (13.54)
Widowed	51.00 (7.07)	> 10	53.20 (14.40)
	*p* = 0.01**		*p* = 0.36**
Education level		Position	
Bachelor's degree	53.83 (13.20)	Clinical nurse	50.57 (11.80)
Master's degree	54.76 (11.71)	Shift supervisor	61.92 (7.92)
Doctorate degree	50.70 (11.27)	Head nurse	71.90 (10.72)
	*p* = 0.70**		*p* < 0.001**
Parental status		Unit	
Not applicable (single)	56.60 (12.26)	Medical surgical unit	51.39 (12.53)
With children	56.43 (13.57)	Intensive care units	62 (12.14)
Without children	53.83 (11.32)	Emergency unit	50.44 (11.57)
	*p* = 0.04**		*p* < 0.001**
Housing status		Average patient‐to‐nurse ratio in unit	
Owned	54.07 (13.51)	< 3	64.00 (12.96)
Rented	57.00 (12.09)	3 to 4	61.26 (11.73)
Living with parents	50.01 (11.70)	5 to 8	53.89 (11.62)
	*p* = 0.006**	> 8	48.45 (11.65)
			*p* < 0.001**
Insurance status		Incentive bonus	
Insured	53.73 (13.09)	Yes	58.02 (11.11)
Uninsured	55.88 (9.26)	No	47.49 (13.04)
	*p* = 0.62*		*p* < 0.001*
Shift pattern		Income	
Fixed	58.30 (11.06)	Less than average	34.33 (4.44)
Rotating	53.17 (13.12)	Average	56.42 (8.45)
	*p* = 0.03*	Higher than average	73.90 (8.83)
			*p* < 0.001**
Age		Monthly working hours	
*r* = −0.007	*p* = 0.90***	*r* = 0.02	*p* = 0.65***

* Independent *T*‐test.

** One‐way ANOVA.

*** Pearson correlation.

One‐way analysis of variance (ANOVA) was used to explore the relationship between polytomous categorical variables and work excitement. Significant differences were found for marital status, parental status, housing status, job position, unit, patient‐to‐nurse ratio, and income level. Post hoc pairwise comparisons revealed specific differences:

- Marital status: Married individuals reported higher work excitement compared to single individuals (*p* = 0.02).
- Parental status: Individuals with children reported higher work excitement than those without children (*p* = 0.004).
- Housing status: Nurses living in rented accommodations reported higher work excitement than those living with their parents (*p* = 0.007).
- Job position: Head nurses reported the highest work excitement, followed by shift supervisors and then staff nurses (all *p* < 0.05).
- Unit: Nurses working in intensive care units reported higher work excitement compared to those in medical/surgical or emergency units (all *p* < 0.05).
- Patient‐to‐nurse ratio: Work excitement decreased as the patient‐to‐nurse ratio increased, with the exception of the comparison between nurses responsible for fewer than two patients and those responsible for 3–4 patients (*p* = 0.83).
- Income: Nurses with higher incomes reported higher levels of work excitement (all *p* < 0.05).

Pearson correlation analysis was used to examine the relationship between continuous variables and work excitement. No significant correlations were found between age and monthly working hours and work excitement (*p* > 0.05). Based on these findings, Hypothesis 1 was supported.

### 3.3 Hypothesis 2

There is a relationship between individual and occupational factors of nurses and their level of work frustration.

The mean work frustration score for all participants was 61.77 (SD = 11.10). As shown in Table 3, independent samples t‐tests revealed significant associations between shift pattern and receipt of incentive bonuses and work frustration (*p* < 0.05). Nurses working fixed shifts and those receiving incentive bonuses reported lower levels of work frustration.

**TABLE 3:** Work frustration scores and their relationship to demographic and occupational factors.

Variables	Mean (SD)	Variables	Mean (SD)
Gender		Employment status	
Male	60.34 (11.04)	Permanent	61.96 (11.89)
Female	62.86 (11.05)	Temporary	61.50 (9.91)
	*p* = 0.06*		*p* = 0.74*
Marital status		Work experience	
Single	64.82 (10.43)	< 1	66.33 (10.29)
Married	59.94 (11.17)	1 to 5	60.88 (9.97)
Divorced	59.11 (11.27)	6 to 10	61.59 (11.66)
Widowed	66.00 (5.65)	> 10	62.18 (11.83)
	*p* = 0.005**		*p* = 0.36**
Education level		Position	
Bachelor's degree	61.77 (11.24)	Clinical nurse	64.44 (10.13)
Master's degree	60.84 (10.27)	Shift supervisor	55.39 (7.92)
Doctorate degree	64.10 (10.40)	Head nurse	46.20 (7.92)
	*p* = 0.735**		*p* < 0.001**
Parental status		Unit	
Not applicable (single)	64.82 (10.43)	Medical surgical unit	63.01 (10.56)
With children	61.25 (10.11)	Intensive care units	54.04 (9.98)
Without children	59.45 (11.45)	Emergency unit	65.46 (9.81)
	*p* = 0.001**		*p* < 0.001**
Housing status		Average patient‐to‐nurse ratio in unit	
Owned	61.32 (11.40)	< 3	51.87 (9.31)
Rented	59.06 (10.74)	3 to 4	55.17 (10.23)
Living with parents	65.23 (10.00)	5 to 8	60.54 (10.04)
	*p* = 0.006**	> 8	67.57 (9.16)
			*p* < 0.001**
Insurance status		Incentive bonus	
Insured	61.79 (11.19)	Yes	58.38 (10.06)
Uninsured	61.11 (8.37)	No	66.84 (10.68)
	*p* = 0.85*		*p* < 0.001*
Shift pattern		Income	
Fixed	57.57 (9.22)	Less than average	77.20 (2.36)
Rotating	62.37 (11.23)	Average	59.87 (8.21)
	*p* = 0.02*	Higher than average	44.19 (4.95)
			*p* < 0.001**
Age		Monthly working hours	
*r* = −0.012	*p* = 0.84***	*r* = −0.004	*p* = 0.94***

* Independent *T*‐test.

** One‐way ANOVA.

*** Pearson correlation.

One‐way ANOVA indicated significant differences in work frustration based on marital status, parental status, housing status, job position, unit, patient‐to‐nurse ratio, and income level. Post hoc pairwise comparisons showed:

- Marital status: Single individuals reported higher levels of work frustration compared to married individuals (*p* = 0.009).
- Parental status: Single individuals reported higher levels of work frustration compared to those with children (*p* = 0.001).
- Housing status: Individuals living with family reported the highest levels of work frustration, followed by those living in rented accommodations and those with their own homes (all *p* < 0.05).
- Job position: Head nurses reported the lowest levels of work frustration, followed by shift supervisors and then staff nurses (all *p* < 0.05).
- Unit: Nurses working in intensive care units reported lower levels of work frustration compared to those in medical/surgical or emergency units (all *p* < 0.05).
- Patient‐to‐nurse ratio: Work frustration increased as the patient‐to‐nurse ratio increased, with the exception of the comparison between nurses responsible for fewer than two patients and those responsible for 3–4 patients (*p* = 0.60).
- Income: Nurses with higher incomes reported lower levels of work frustration (all *p* < 0.05).

Pearson correlation analysis did not reveal significant correlations between age and monthly working hours and work frustration (*p* > 0.05). Based on these findings, Hypothesis 2 was supported.

### 3.4 Hypothesis 3

Nurses' levels of work excitement and work frustration are inversely related.

**FIGURE 1:**
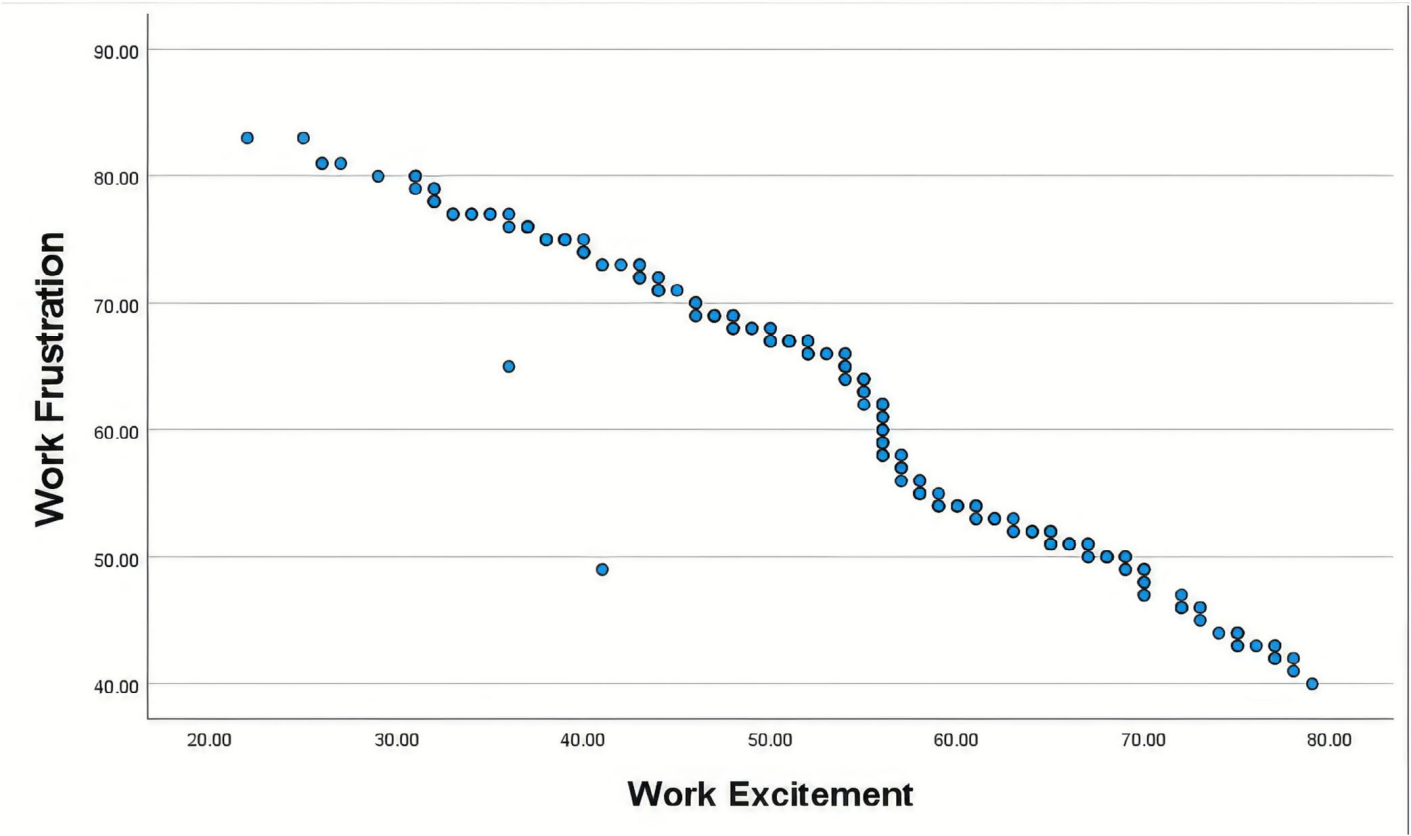
Scatterplot illustrating the negative correlation between work frustration and work excitement among nurses.

A Pearson correlation analysis revealed a significant negative correlation (*r* = −0.97, *p* < 0.001) between work excitement and work frustration, supporting Hypothesis 3. This inverse relationship is graphically depicted in Figure 1.

## 4 Discussion

The present study findings presented a strong negative correlation between work excitement and work frustration, suggesting that factors diminishing work excitement often contribute to increased job frustration and vice versa. A straightforward explanation for this inverse relationship can be found in the definitions of these two variables.

While providing a precise definition for the subjective experience of work excitement is challenging, it can be conceptualised as an intrinsic motivation and passion experienced in the workplace (Chang et al. 2019). Conversely, work frustration refers to the negative emotions experienced by employees due to perceived barriers in achieving work‐related goals and needs (Lin et al. 2019). These definitions suggest that the two variables represent nearly opposite ends of the emotional spectrum experienced by employees in the workplace, thus justifying the negative correlation. Chang et al.'s (2019) research aligns with these findings, also demonstrating a negative correlation between the two variables.

Halcomb and Ashley's (2017) study further supports this relationship by showing that organisations providing employees with opportunities to achieve their goals (reducing work frustration) tend to have employees with higher levels of accomplishment and work excitement. This indirectly corroborates the inverse relationship between work frustration and work excitement.

However, Bruffey's (1993) earlier study on factors influencing nurse retention revealed a discrepancy in these findings. Despite high levels of work excitement, many nurses reported high work frustration and low job satisfaction. This inconsistency might be attributed to the extremely low job satisfaction among the nurses in Bruffey's study. It can be inferred that even with high work excitement, work frustration can be elevated in situations of extremely low job satisfaction. Further research is needed to explore this possibility.

### 4.1 Occupational Factors Influencing Work Excitement and Work Frustration Among Nurses

The present study revealed that various occupational factors, such as financial considerations (income and incentives), caring for fewer patients (less than four), working in intensive care units, holding higher positions (head nurse and shift supervisor), and working fixed shifts, were associated with increased work excitement and decreased job frustration among nurses. Conversely, factors such as years of experience, monthly working hours, and employment status did not show a significant correlation with work excitement or work frustration. Considering that previous research has linked work excitement and work frustration to organisational commitment (Chang et al. 2019, 2014) and burnout (Gribben and Semple 2021), respectively, it can be inferred that the variables correlated with work excitement and work frustration in the present study may also influence nurses' organisational commitment and burnout.

Pallavi et al.'s (2023) study indicated that flexible timing can increase work excitement among employees. Similarly, in the present study, nurses who had more flexibility in their work schedules and worked fixed shifts reported higher levels of work excitement. This suggests that having control over work timing is important for employees and can positively impact work excitement. Wang et al.'s (2016) research further demonstrated that sleep disturbances can increase feelings of work frustration among nurses, which aligns with the findings of the present study highlighting the importance of fixed shifts to ensure more consistent and restful sleep patterns, ultimately enhancing nurses’ work performance and decreasing work frustration.

Consistent with previous studies, the present study found that nurses working in intensive care units experienced lower levels of job frustration. Chang et al. (2019) showed that job variety and learning opportunities can increase work excitement. Based on these findings, it was expected that emergency care nurses would have the highest levels of work excitement, followed by nurses in intensive care units and then medical‐surgical units. However, the results of the present study indicated that nurses in intensive care units had the highest levels of work excitement, while emergency room nurses and medical‐surgical unit nurses had relatively similar levels. This discrepancy can be attributed to the exceptionally high number of patients under care in emergency departments during critical periods such as the COVID‐19 pandemic, as the present study demonstrated that the number of patients under care impacts work excitement, with higher numbers leading to decreased work excitement.

Xuan and Vy's (2023) research indicated that factors such as career opportunities and individuals' perceptions of their role as nurses can enhance their work excitement. These findings may justify the results of our study, which show that nurses in higher positions exhibit greater work excitement. This could be attributed to their better access to career opportunities and a clearer understanding of their professional roles. Additionally, the more defined and specialised roles of nurses in specialised units may be another factor contributing to higher levels of work excitement and lower levels of work frustration among nurses in these units.

In a qualitative study by Jannah and Hernawan (2023), factors such as ambiguous roles and responsibilities, lack of support and recognition, organisational injustice, lack of opportunities for career growth, and work‐life imbalance were identified as contributing to work frustration. Based on these findings, one possible reason for higher levels of work frustration among nurses in lower positions in the present study could be due to a lack of sufficient recognition and limited opportunities for career growth, whereas nurses in higher positions, such as head nurses, may receive more recognition and have a better sense of career progression.

Murkhana et al.'s (2023) study demonstrated that increased workload and disruptions in work‐life balance can be related to increased work frustration. This finding aligns with the results of the present study, which showed that a higher number of patients under care (increased workload) and irregular shifts (disruption in work‐life balance) contributed to higher levels of work frustration among nurses.

### 4.2 Individual Factors Influencing Work Excitement and Work Frustration Among Nurses

Our results indicated that being married, having children, and owning a home were positively associated with work excitement and negatively associated with work frustration. Male nurses reported higher levels of work excitement and lower levels of work frustration compared to female nurses, although this difference approached but did not reach statistical significance (*p* = 0.7 for work excitement and *p* = 0.6 for work frustration). Additionally, while not statistically significant, nurses with doctoral degrees reported lower levels of work excitement and higher levels of work frustration compared to nurses with lower academic degrees. Age was not found to be significantly related to either work excitement or work frustration.

Although previous research has shown that work–family conflict can increase work frustration (Murkhana et al. 2023), and married individuals may face greater challenges in balancing work and family responsibilities, the current study found that married nurses reported lower levels of work frustration. This finding contradicts the results of most previous studies. For example, Okayasu et al.'s (2022) study found that married nurses experienced lower work‐life balance, which could ultimately lead to increased work frustration. Similarly, Aziz and Aman‐Ullah (2023) study showed that being married can increase the risk of work‐life imbalance. Despite these discrepancies, the unique context of the COVID‐19 pandemic, during which the study was conducted, provides a potential explanation. The demanding work conditions during the pandemic may have increased nurses’ need for emotional support from their families (Hidayati et al. 2022; Lubaba and Ediati 2022), particularly for married nurses and those with children. This increased family support could have mitigated the negative impacts of work–family conflict and contributed to lower levels of work frustration among married nurses.

Owning a home, often associated with higher income, can provide financial stability. In this study, both homeownership and higher income were positively linked to work excitement and negatively linked to work frustration, consistent with previous research. For instance, Mukhtar et al. (2022) found that financial stability can positively impact work‐life balance, indirectly reducing work frustration. Moreover, numerous studies have confirmed the positive relationship between financial rewards and job satisfaction and performance which can enhance work excitement and reduce work frustration (Chi et al. 2023; Kim et al. 2022). Considering the financial challenges faced by many individuals, particularly in countries with high inflation and economic instability, addressing financial concerns can significantly improve nurses' job performance and reduce work frustration.

Regarding the higher levels of work frustration among nurses with doctoral degrees, while there is limited comparable research, one possible explanation is that these individuals may have higher expectations of their jobs. With advanced academic qualifications, they may anticipate more opportunities for research and scholarly activities. However, the clinical demands of nursing often limit such opportunities, leading to feelings of underutilisation and frustration. Further research is needed to explore the specific expectations of doctoral‐prepared nurses working in clinical settings and to develop strategies to address these expectations.

## 5 Implications for Policy and Practice

Our results suggest several concrete actions healthcare managers and policymakers can take.

First, consider targeted financial incentives (e.g., hazard/incentive bonuses or overtime premiums) to acknowledge increased burden and improve morale. Second, prioritise staffing interventions to reduce patient‐to‐nurse ratios (e.g., temporary redeployment, surge staffing pools, or accelerated hiring) because lower caseloads were associated with greater work excitement and lower frustration. Third, revise scheduling practices to increase predictability and worker control—for instance, offer fixed or self‐rostering options and limit consecutive long shifts—to support sleep and work–life balance. Fourth, invest in non‐financial supports: structured continuing education, clear career progression pathways (to retain and motivate nurses in higher roles), and on‐site or easily accessible mental‐health and peer‐support services. Finally, implement monitoring (regular staff surveys, turnover and sickness metrics) and pilot test any major changes locally to evaluate impact before scale‐up. These relatively low‐cost, evidence‐aligned measures can mitigate frustration and strengthen work excitement, ultimately supporting quality of care during and after pandemic conditions.

## 6 Limitation

While this study provides valuable insights into the factors influencing work excitement and frustration among nurses, several limitations should be acknowledged.

Firstly, the cross‐sectional design of the study limits the ability to establish causal relationships between variables. Furthermore, the sample was drawn from a specific geographical region, which may limit the generalisability of the findings to other healthcare settings. Finally, the self‐report nature of the data collection method may be susceptible to social desirability bias.

## 7 Conclusion

The COVID‐19 pandemic has significantly impacted the mental and emotional well‐being of healthcare workers, particularly nurses. This study sheds light on the factors influencing work excitement and frustration among nurses during this challenging period. A strong negative correlation between these two emotions underscores the importance of addressing both positive and negative aspects of the work environment to mitigate the detrimental effects of the pandemic on nurses' mental health.

Occupational factors, such as excessive workload, irregular shift patterns, and inadequate staffing levels, exacerbated by the pandemic, have contributed to increased work frustration among nurses. Conversely, factors like flexible work schedules, opportunities for professional development, and adequate financial compensation have been associated with higher levels of work excitement.

Individual factors, such as marital status, family responsibilities, and housing situations, have also played a role in shaping nurses' emotional experiences during the pandemic. While some factors, such as family support, have provided a buffer against stress, others, such as financial strain and isolation, have exacerbated feelings of frustration.

Further research is needed to explore the long‐term implications of work excitement and work frustration on nurse well‐being, patient outcomes, and organisational performance. By understanding the underlying factors that influence these emotions, healthcare organisations can create a more supportive and motivating work environment for nurses. Implementing targeted staffing, scheduling, financial, and psychosocial supports may help healthcare organisations improve nurses' work excitement and reduce work frustration, thereby enhancing workforce resilience and patient care.

## Author Contributions

**Alireza Houshangi:** research implementation, research design, and article review. **Ali Mohammadabadi:** research design, article writing, translation, and review. **Fateme Noghani:** assistance with article design and review. **Shima Haghani:** Statistical analysis and article review.

## Funding

The authors wish to acknowledge funding support for this study from Tehran University of medical sciences.

## Ethics Statement

Tehran University of Medical Sciences, IR.TUMS.FNM.REC.1400.048.

## Conflicts of Interest

The authors declare no conflicts of interest.

## Data Availability

The datasets generated during and/or analysed during the current study are available from the corresponding author on reasonable request.
